# Cenh3: An Emerging Player in Haploid Induction Technology

**DOI:** 10.3389/fpls.2016.00357

**Published:** 2016-04-12

**Authors:** Anne B. Britt, Sundaram Kuppu

**Affiliations:** Department of Plant Biology, University of California, DavisDavis, CA, USA

**Keywords:** uniparental inheritance, Centromere, plant breeding, CENH3, haploid induction

## Abstract

True-breeding lines are required for the development and production of crop varieties. In a classical breeding approach these lines are obtained through inbreeding, and often 7–9 generations of inbreeding is performed to achieve the desired level of homozygosity, over a period of several years. In contrast, the chromosomes of haploids can be doubled to produce true-breeding lines in a single generation. Over the last century, scientists have developed a variety of techniques to induce haploids and doubled haploids, though these techniques apply only to particular crop varieties. Ravi and Chan (2010) discovered that haploids could be obtained in *Arabidopsis* through the manipulation of the centromere-specific histone 3 variant, CENH3. Their approach, which involved extensive modifications to a transgenic CENH3, held promise of being translated to crop species, and has been successfully employed in maize (see Kelliher et al., 2016). Refinements of this technology have since been developed which indicate that non-transgenic modifications to CENH3 will also induce haploids. The complementation of a *cenh3* null by CENH3 from closely related plant species can result in plants that are fertile but haploid-inducing on crossing by *CENH3* wt plants- suggesting that introgression of alien CENH3 may produce non-transgenic haploid inducers. Similarly, a remarkably wide variety of point mutations in CENH3, inducible by chemical agents, have recently been shown to result in haploid induction on crossing by wild-type CENH3 plants. These CENH3-variant plants grow normally, are fully fertile on self-pollination, and may be present in existing mutagenized collections.

## The Variant Histone Cenh3 Is Required For Centromere Localization

Eukaryotic DNA is wrapped around nucleosomes. This wrapping, and subsequent orders of supercoiling of the nucleosome/DNA complex, serves to compact the chromosome, allowing the rather long linear genome in flowering plants, between 61 Mb (*Genliseatuberosa*) (Fleischmann et al., 2014) and 160 Gb (*Paris japonica*) (Pellicer et al., 2010) to fit into a rather small compartment- the nucleus of the cell. Compaction of the entire genome or discrete sections within it varies with both the stage of the cell cycle and the function of the DNA. The degree of supercoiling of any particular region of the chromosome is determined by the presence or absence of particular histone variants that make up each nucleosome and by posttranslational modifications of these variants (Gibcus and Dekker, 2013; Cutter and Hayes, 2015). The pattern of histone deposition and modification is maintained after DNA replication, and in some cases across generations, through a variety of mechanisms (Mattiroli et al., 2015). This semi-stable modification of polymerase access through the modification of nucleosomal histone content, often heritable as a pseudoallele, has come to be referred to as “epigenetic” regulation of gene expression and heritable forms of this modification are termed epigenetic inheritance.

An exceptional example of epigenetic determination of chromatin function is the role of histone content in the localization of the centromere. While centromeric DNAs in eukaryotic species often share general features (long arrays of tandem repeats, often interspersed with transposable elements) recent findings have demonstrated that these features are not essential to centromere localization; some eukaryotes lack long tandem arrays entirely, some have different repeat arrays on different chromosomes, and neocentromeres can form at unique sequences (Marshall et al., 2008). Rather than being determined by DNA sequence the location of the centromere is thought to be determined by the presence of nucleosomes carrying the variant histone CENH3 (Allshire and Karpen, 2008; Stimpson and Sullivan, 2010; Henikoff and Smith, 2015; Westhorpe and Straight, 2015; also known as CID in *Drosophila*, CENP-A in mammals and Cse4 in yeast; Talbert et al., 2002; Sekulic and Black, 2012; Lermontova et al., 2014; Steiner and Henikoff, 2015), although in some exceptional cases (notably *Saccharomyces cerevisiae*) this histone variant does have sequence-specific DNA binding affinity (and so centromeres will form at specific DNA sequences). Thus in the majority of eukaryotes studied, centromere positioning appears to be an epigenetic, rather than sequence-based, phenomenon (Liu et al., 2015).

CENH3, like other histones, carries an N-terminal tail (which protrudes from the nucleosome and is a target for posttranslational modification) and C-terminal Histone Fold Domain (which interacts with DNA and other histones to form the nucleosome). Unlike conventional histones, CENH3 is rapidly evolving. The N-terminal tail of CENH3 barely alignable even among closely related species whereas the histone fold domain is relatively well conserved (Malik and Henikoff, 2003).

Because CENH3 determines the position of the centromere, the presence of CENH3 is essential for segregation of chromosomes. Alteration of CENH3 in several organisms has been shown to have adverse consequences leading to chromosome segregation errors and lethality. In budding yeast mutations in *CENH3* cause chromosome non-disjunction and cell cycle arrest at mitosis (Stoler et al., 1995). In *Caenorhabditis elegans*, knockout of CENH3 induces missegregation and lethality (Buchwitz et al., 1999). In *cenpA* null mice, early disruption of centromeric chromatin organization has been observed (Howman et al., 2000). Reduction in the level of CENPA in human cell lines induces p53-dependent cellular senescence (Maehara et al., 2010), presumably to prevent cells from undergoing error-prone mitosis. Down-regulation of *CENH3* in *Arabidopsis* leads to reduced mitosis and increased meiotic segregation errors (Lermontova et al., 2011). Homozygous *cenh3*-null mutants of *Arabidopsis* exhibit early embryonic lethality (Ravi et al., 2010).

Given the importance of maintaining one- and only one- centromere per chromosome, the loading of CENH3-containing nucleosomes at a single locus per chromosome is a critical step in genome maintenance. As we will discuss below, haploid induction (HI) in CENH3-modified lines may result from errors in reloading of CENH3 at centromeres derived from CENH3-modified lines. Although the reloading process has been extensively investigated in animals and fungi (Müller and Almouzni, 2014), we are only beginning to learn about this process in plants (Lermontova et al., 2013; Liu et al., 2015).

## The Importance of Haploid Induction

Doubled haploids- plants carrying a genome derived from a single (haploid) gamete- provide enormous timesaving in the production of true-breeding lines, literally reducing the time to acceptable levels of homozygosity by an order of magnitude. Haploid lines, and “haploid genetics” can accelerate a variety of genetic constructions and is useful in the research laboratory also (Ravi et al., 2014). The spontaneous occurrences of haploids have been reported in several species reviewed in (Dunwell, 2010; Dwivedi et al., 2015). In some species haploids have been generated through interspecies and intraspecies hybridization. (Guha and Maheshwari, 1964) showed that haploid plants could be generated from anther culture in some plant species. A variety of other protocols have been developed to generate haploids, reviewed in detail in Wędzony et al. (2009). Though all of these techniques have proved to be useful over the last 50 years, they are limited to few crop species and/or varieties. In this review we will highlight the recent advances in HI technology via modification of CENH3. Because CENH3 is present in all plants there is promise that this technology can be translated to the majority of crop species.

## CENH3 Mediated Haploid Induction

**FIGURE 1:**
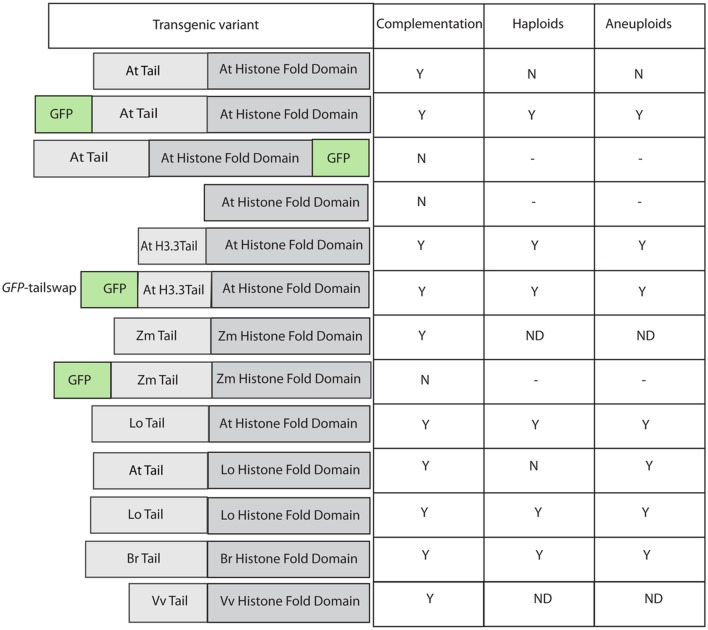
**Effect of various modifications of transgenic *CENH3* variants in an *Arabidopsis thaliana cenh3* null.** Table on the right summarizes complementation (of viability and fertility), haploid, and aneuploid induction by these lines. *GFP-tailswap* is the commonly used nomenclature in published literature for this construct.

In a breakthrough discovery, Ravi and Chan (2010) studying the structure/function relationship of Histone 3 variants, found that a *cenh3*–/– *Arabidopsis* null mutant, when complemented by altered version of *CENH3*, could induce haploids. A remarkably effective haploid inducer, termed “green fluorescent protein (*GFP)-tailswap*,” carried GFP fused to the N-terminal tail domain of an H3 variant (At1g13370), which then replaced the N-terminal tail of CENH3 (see **Figure 1**). They found, surprisingly, that the CENH3 mitotic functionality was maintained in spite of the “tailswap,” though meiotic function was compromised- the plants were almost entirely male sterile, with some female sterility also. On self-pollination, *GFP-tailswap* produces normal diploid seeds (though again, with reduced fertility). However, when crossed as a female by plants carrying wild-type CENH3, *GFP-tailswap* produces a remarkable 25–45% paternal haploid progeny. The maternal (*GFP-tailswap*) genome is lost in these haploids (Ravi and Chan, 2010). The almost total male sterility observed in *GFP-tailswap* plants is ascribed to its meiosis-specific loading abnormalities (Ravi et al., 2011). The authors also observed some, though less frequent, HI in plants carrying a simple N-terminal addition of GFP to CENH3 (no “tailswap”). The three independently derived lines of *GFP-tailswap* analyzed did not display significant differences in their level of HI frequencies (Tan et al., 2015). This suggests that HI is dependent on the modification of CENH3 rather than on expression levels- although it must be noted that a certain minimum, and perhaps maximum (Marshall et al., 2008) level of expression is selected for in these transgenic experiments, which are performed in a *cenh3–/–* background.

The initial findings with *GFP-CENH3* and *GFP-tailswap* suggested that addition of a bulky tag to CENH3 might interfere with CENH3 recognition, and so produce a centromere that is less competitive for the reloading of CENH3 or subsequent components of the centromere when confronted with competing wild-type centromeres. Working from this hypothesis, we will refer to these modified CENH3’s, and their resulting centromeres, as “weak” or “non-competitive” vs. wild-type CENH3. Addition of GFP on its own presumably results in a slightly defective protein, and modification of the N-terminal tail (tailswap) further degrades the strength of the resulting centromere.

The addition of adducts such as GFP, and changes in the sequence of CENH3, can act additively to affect protein function. For example, as described above, GFP-CENH3, in *Arabidopsis*, is a haploid inducer, but otherwise fully functional. The *Arabidopsis* null mutant can also be complemented (mitotically and meiotically) by CENH3 from a wide range of angiosperms (Maheshwari et al., 2015), including the monocot *Zea mays*. However, addition of GFP to the N-terminus of this distantly related protein compromised its function to the extent that while it could localize to centromeres, it could not complement the lethality of the *cenH3–/–* mutant (Ravi et al., 2010; **Figure 1**).

## CENH3 Function is Conserved Across Wide Variety of Species

In pioneering study with human cell lines, it was shown that yeast *CENH3* (*Cse4*) was able to complement a human defect in *CENH3* (*CENPA*) function (Wieland et al., 2004). Similarly, Moraes et al. (2011), found that EYPF-tagged versions of CENH3 from monocentric plant species like, *Arabidopsis lyrata, A. arenosa, Capsella bursa-pastoris*, and *Z. mays* were able to target the *A. thaliana* centromere. In contrast, an EYPF tagged version of *CENH3* from holocentric plant *Luzula nivea* failed to target *A. thaliana* centromeres. Ravi et al. (2010) found that GFP-tagged versions of CENH3 from *A. aeronosa, A. lyrata*, and *Z. mays* were able to target *A. thaliana* centromere. However, the authors also found that GFP-tagged versions of *CENH3* from other kingdoms (*S. cerevisiae, C. elegans* and *Homo sapiens*) did not target to *A. thaliana* centromeres. In the same study it was shown that GFP tagged versions of CENH3 from *Brassica rapa* and *Z. mays* were able to localize to *A. thaliana* centromere, but were unable to complement *A.thaliana cenh3* null function. This result was surprising given ability of the yeast protein to complement the human defect, which suggests that CENH3 function is highly conserved across kingdoms. This discrepancy was explained when Maheshwari et al. (2015), in a subsequent study employed untagged versions of CENH3 from *B. rapa*, *Lepidium oleracuem, Vitis Vinifera*, and *Z. mays* found that all of these untagged proteins were able to complement *A. thaliana cenh3* null function. In another intriguing note, when the GFP is attached C-terminally to the *AtCENH3* protein, it is able to localize to the centromere but unable to complement *cenh3* null function in *Arabidopsis* (Ravi et al., 2010), while the N-terminally tagged protein restores viability and full fertility to this mutant. Thus GFP-tagging of CENH3 often places restrictions on its functionality. From the above studies using heterologous, untagged CENH3, it’s clear that the essential functions of *CENH3* are well conserved across a wide variety of species.

## A Role for N-Terminal Domain Mutations in Haploid Induction

**FIGURE 2:**
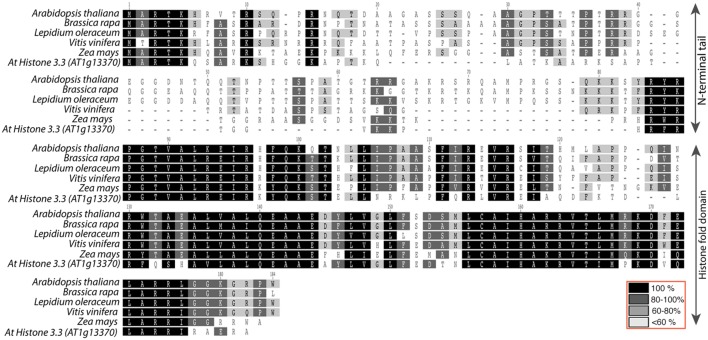
**Alignment of *Arabidopsis thaliana* histone3.3 vs. CENH3 sequences from *A. thaliana, Brassica rapa, Lepidium oleraceum, Vitis vinifera*, and *Zea mays*.** Top two rows represent N-terminal tail with less conservation and the bottom three columns represent relatively well-conserved histone fold domain.

The Ravi and Chan (2010) study described above not only indicated that substantial alterations to CENH3 can result in HI, it also demonstrated, surprisingly, that the N-terminal domain of CENH3 is not required for its mitotic function. In a study to designed to determine whether naturally occurring divergence in CENH3 sequence can result in functional constraints, Maheshwari et al. (2015) found that CENH3 function is well conversed across species on a broad evolutionary scale. *Arabidopsis cenh3-1* mutants transformed with exotic *CENH3*s from *B. rapa, L. oleraceum, V. vinifera*, and *Z. mays* were viable and fertile. However, although these CENH3s are fully functional in terms of mitosis, meiosis, and in self-pollinating crosses, chromosomes derived from lines expressing the alien but closely related CENH3s from *B. rapa* and *L. oleraceum* proved non-competitive when crossed by wild-type plants, producing aneuploid and haploid offspring that retained the wild-type parent’s genome. Furthermore, the authors demonstrated that replacement of the *Arabidopsis* N-terminal tail with the N-terminal tail from *L. oleraceum* produced plants that, while fully viable and fertile on self-pollination, exhibited extensive seed death, as well as induction of haploidy and aneuploidy, when crossed by wild-type lines. The reverse tailswap, carrying the At-NTD and the LoHFD, did not induce haploids at the scale tested (approximately 100 progeny) though some aneuploids were reported. Thus the sequence of the N-terminal tail affects the “strength” of CENH3 in a competitive cross. The difference in sequence between the N-terminal tails of *L. oleraceum* and that of *A. thaliana* is substantial- they are of similar lengths but differ at 29/82 positions (see alignment in **Figure 2**). Similarly, the N-terminal tail of histone H3.3 (see alignment in **Figure 2**) has very little similarity to that of CENH3, but it complements CENH3’s mitotic and meiotic functions and is, in the absence of the GFP tag, a weak haploid inducer. Whether subtle differences in the N-terminal tail- for example, single amino acid substitutions- can possibly affect the “strength” of CenH3 remains to be determined.

## Point Mutations in CENH3 Histone Fold Domain Induce Haploids

The tail region of CENH3 is rapidly evolving in both length and sequence whereas the histone fold domain is well conserved. This suggests that subtle changes in the HFD might have substantial effects on its activity. Recently, it has been shown that point mutations resulting in single amino acid substitutions induced uniparental genome elimination, resulting in haploidy or aneuploidy, with retention of the wild-type chromosomes (Karimi-Ashtiyani et al., 2015; Kuppu et al., 2015). Remarkably, changes in the majority of conserved residues tested resulted in plants, which were viable, fertile, and, to various degrees, haploid-inducing. None of the tested residues were essential to mitotic or meiotic function. The single amino acid substitutions P82S, G83E, A86V, A132T, L130F, and A136T (*Arabidopsis* CENH3) induced haploids (among 0.6–12 percent of progeny, depending on the mutation), while P102S and G173E appeared to be fully competitive with wild-type CENH3. Aneuploid progeny were also obtained from among the progenies of these plants. Because these mutations could be obtained in other crop plants by ethyl methane sulfonate (EMS) mediated mutagenesis (the sites tested were conserved among crops), this discovery suggests that non-transgenic haploid inducers could be induced- and may already exist, undetected- in EMS mutagenized crop species.

## Shattered Chromosomes and Minichromosomes are Produced During CENH3- Mediated Genome Elimination

Genome loss presumably results from the inability of one parent’s chromosomes to efficiently attract reloading of CENH3 and/or additional constitutive centromeric components; either of these defects would result in a compromised ability to attract outer kinetochore components and a failure to attach to the spindle. The mixed haploid, diploid, and aneuploid progeny of CENH3-based HI crosses indicates that the individual HI-derived chromosomes have independent fates. In some cases, individual chromosomes derived from HI crosses appear to have undergone substantial rearrangements, duplications and/or truncations. These events have been particularly well characterized in progeny of *GFP-tailswap* crosses in *Arabidopsis* (Tan et al., 2015). The authors of this study employed low-pass whole-genome sequencing to bin and count genomic sequences from hundreds of progeny of an HI × wt cross. This method allows the researcher to detect imbalances in copy number (balanced translocations are not detectable via this procedure). The great majority of the plants characterized were chosen from among the 37% of all progeny that resembled previously characterized (Steinitz-Sears, 1963) “numerical” aneuploids- plants carrying a diploid genome plus one or more entire additional chromosome.

Analysis of these putative aneuploids revealed that plants carrying 2*n* + 1 (or more) chromosomes were indeed the most commonly encountered class of aneuploids. Because the analysis was sequence-based, and the crosses were between two different ecotypes, the parental origin of each chromosome could also be scored. In all cases the supernumerary chromosomes were derived from the *GFP-tailswap* (here also female) parent. The second most commonly encountered class of aneuploids were diploids carrying an additional truncated chromosome (again *GFP-tailswap*-derived). In a very small fraction of aneuploids, during the process of genome elimination, a chromosome from the *GFP-tailswap* parent undergoes “shattering,” revealed by copy number discrepancies within a single supernumerary chromosome. The investigators observed the encapsulation of chromatin into micronuclei, and hypothesize that one or more laggard *GFP-tailswap* chromosomes left behind during segregation can be compartmentalized into micronuclei, which occasionally rejoin the nucleus proper. At some point during this excursion, the chromosomes undergo endonucleolytic degradation (referred to as “shattering”) and subsequent rejoining, resulting in variety of intra- or interchromosomal rearrangements. Retention of– or fusion to- a centromeric region would result in heritability. Meiotically heritable mini- and interchromosomal rearrangements were observed and confirmed cytologically. The resulting mini-chromosomes are potentially of value for plant breeding, if they can be designed to carrying a one or more useful traits in a single, simple package that can be easily transferred between varieties.

## Recombination of Shattered Chromosomes Occurs Via Non-Homologous End Joining (NHEJ)

The source of the double strand breaks that result in shattered chromosomes is unclear. Breaks might be induced by prolonged exposure to the cytoplasmic environment, or through the deregulation of replication vs. repair in micronuclei. Rearrangements could theoretically be generated either via non-homologous end joining or through ectopic homologous recombination (HR) of repetitive DNAs. Tan et al. (2015) cloned and sequenced 12 randomly selected junctions derived from a single shattered chromosome, and found that all displayed junction characteristic of NHEJ events, including the presence of microhomologies at the joint and the insertion of sequences of unknown origin. The authors then went on to investigate the effects of deficiencies in DNA ligase IV, an enzyme routinely involved in NHEJ. Crossing *GFP-tailswap* by plants homozygous for a null mutation in *Lig4* resulted in an increased frequency of haploids (doubling from 40% of progeny to 80%) at the expense of both diploid and aneuploid progenies. This suggests that *both* aneuploids *and* diploids experience *LIG4*-dependent chromosome rescue. This in turn suggests that the *GFP-tailswap*-derived chromosomes in diploids may have undergone extensive rearrangement and/or point-like mutations that are undetectable via the phenotypic screen employed to collect aneuploids for sequence-based characterization.

Fascinatingly, the *LIG4* status of the *GFP-tailswap* (maternal) parent has no effect on HI frequency; the observed *LIG4*-dependent rescue of aneuploids and diploids depended entirely on the (wild-type *CENH3*) paternal genome. A potential explanation for this phenomenon is that chromosomes- even chromosomes that will eventually produce diploids- are frequently being rescued into what is essentially a haploid, paternal nucleus, and that the (perhaps extranuclear) *GFP-tailswap*-derived genome is not effectively expressed during this process. In mammalian systems, it has also been shown that unrepaired DNA damage increased the frequency of uniparental genome elimination (Wang et al., 2014). This finding clearly shows that DNA repair mutants could be used in CENH3-mediated genome elimination system to enhance the frequency of HI.

## CENH3 Mediated Genome Elimination is Most Efficient When the HI Line is the Female Parent

Three different classes of modifications of CENH3 have been shown to produce haploids: Domain swapping with addition of fluorescent tag, complementing with CENH3 from different species, and point mutations in the highly conserved Histone Fold Domain. It is strikingly evident from all three cases that the HI works well when the CENH3 mutants are used as female parent, but not as a male. In case of *GFP-tailswap* the haploid inducing frequency is around 40 percent when it is used as a female parent, but drops down to 5% when used as a male (Ravi and Chan, 2010). Karimi-Ashtiyani et al. (2015) found that the point mutation L130F induced haploids only used as female parent (at the scale tested). This does not rule out the possibility of using CENH3 mediated HI system as a male, but it is clear that modification of CENH3 has a much stronger haploid-inducing effect when used as a female. The biological significance of this effect is unclear; possibly a higher level of wt CENH3 transcript in the female gametophyte helps to negate the effect of modified CENH3s on loading of additional centromeric factors. However, see below for a discussion of the phenomenology of CENH3 loading and active unloading in the gametophyte and zygote.

## Seed Abortion is Correlated with Haploid Induction in CENH3 Mediated Genome Elimination

A common feature observed in all CENH3 mediated haploid inducing systems is seed abortion (the presence of small, wrinkled, dark, inviable seeds in the silique, amongst plump viable seeds) in the HI cross. All variants of CENH3 (*GFP-tailswap*, CENH3’s from distant species and point mutants) that complement endogenous *cenh3-1* null do not display seed death on self-pollination. Though *GFP-tailswap* is nearly sterile, with poor seed set per silique, those seeds that are produced are plump and healthy. In contrast, when these lines are hybridized to wild-type plants they display substantial seed death, and the frequency of death is positively correlated with the frequency of HI.

In angiosperms, embryogenesis involves double fertilization and triple fusion where one sperm nuclei fuses with the egg to form the zygote and one sperm fuses with two already fused central cells to form the triploid endosperm (a tissue that supports embryonic and seed development). Euploidy of both the zygote and endosperm are important for seed development; defects in chromosomal balance in either tissue type could result in seed death. It makes sense that loss of the HI genome would occur in both the embryo and endosperm, but this is not necessarily true, as CENH3 loading and active unloading rates are developmentally specific (Ingouff et al., 2010). Thus the effects of CENH3 modification on the genome of the endosperm require additional investigation.

In an effort to facilitate screening of haploids at seed level, Ravi et al. (2014) expressed GPF under the control of seed storage protein 2S3 promoter (At2S3: GFP), and transformed this construct into *GFP-tailswap*. This marker is expressed in both embryo and endosperm, and although endosperm tissue is limited in the mature *Arabidopsis* seed, the marker can be visualized through the seed coat of mature, dried seeds in both the seedling (where it is highly expressed) and in the residual endosperm. Seeds derived from *GFP-tailswap* p23S:GFP HI crosses fell into two phenotypic classes: fully GFP+ (endosperm vs. embryonic expression was not distinguished in this class), and mottled GFP expression (in the endosperm, but not the embryo). Mottled seeds always gave rise to haploid (paternal, GFP-) seedlings, while fully green seeds produced diploids or aneuploids. Perhaps the most significant result, aside from the success of the marker for identification of haploid seeds, is that there were no GFP-free seeds, suggesting that fertilization events resulting in paternal “monoploid” endosperm are either very rarely produced (both copies of the HI-inducer’s genome are rarely lost) or generally lead to seed abortion, or particularly lead to abortion of haploids.

## Observation of CENH3 Depletion in the Zygote and Embryo

In a classical barley HI system involving crosses between *Hordeum vulgare × Hordeum bulbosum*, the *H. bulbosum* genome is lost during embryogenesis (Kasha and Kao, 1970). Sanei et al. (2011) were able to cytologically distinguish between the *vulgare* and *bulbosum* genomes via FISH, while simultaneously following deposition of CENH3 via immunolocalization (employing an antibody which does not distinguish between the CENH3s encoded by each species). In their visualization of anaphase mitoses in early embryos, the authors found that lagging chromosomes were, unsurprisingly, *bulbosum*-derived. More notably, these laggards lacked detectable CENH3, which is easily observed via immunolocalization in normal barley chromosomes. This indicates that there is-minimally- a failure of CENH3 to reload onto the *bulbosum* chromosomes, rather than (or perhaps in addition to), a failure of more distal centromeric components to recognize the *bulbosum*-derived centromeres.

It is not clear in the barley system described above whether CENH3 is lost from the *bulbosum* chromosomes due to dilution (with a failure to reload) after replication, or due to active unloading of CENH3 in the zygote. Interestingly, Ingouff et al. (2010) have shown- in *Arabidopsis*- that neither *CENH3* transcripts nor CENH3-GFP (tagged at the C terminus) were detectable in the *Arabidopsis* egg cell. Although the authors observed localization of CENH3-GFP to sperm centromeres, this mark was rapidly lost after fertilization of wild-type eggs, prior to further cell division, in what is apparently the active removal of this mark. Localization of CENH3-GFP was visible later in embryogenesis. This very provocative observation raises the possibility that CENH3 is not present in the zygote. If this were true for CENH3, as well as for CENH3-GFP, this observation would challenge the model that centromeres are determined simply by the presence or absence of CENH3. It remains to be determined, however, whether wild-type CENH3 is similarly actively depleted from the zygote. CENH3-GFP may behave differently than CENH3; it does not, for example, complement the *cenH3–/–* mutation (Ravi et al., 2010).

The observations with point mutants that the behavior of chromosomes in the zygote is determined by the amino acid sequence of the CENH3 protein present in the parental lines- is most easily explained by assuming that significant levels of parental CENH3 are retained in both the gametes and the zygote. However, it is also conceivable that CENH3 is truly absent, and that modification of CENH3 results in some sort of modified- and therefore “weak”- zygotic signal, or “footprint,” at the centromeric locus. It is possible that CENH3 is not effectively reloaded onto these weak footprints.

## Regulatory Issues and Possible Alternatives

In a *CENH3*-mediated HI system, all chromosomes in the haploid product are, from a molecular point of view, non-transgenic, regardless of whether the haploid-inducing parent carried a transgenically modified *CENH3* allele. From a regulatory perspective, however, it is interesting to note that HI through modification of *CENH3* could be considered GMO or non-GMO based on interpretation of the law in the geographical region for which it is being considered. In some parts of the world, the genetically modified organisms are labeled based on the product- the genotype of the plant itself- whereas in other parts GMOs may be identified based on the process employed to generate that plant (Hartung and Schiemann, 2014). *GFP-tailswap* based HI would be considered non-GMO if the product only is taken into account whereas it would be considered GMO if process of generation is an important criterion. Similarly, if CENH3 from other species were introduced through molecular tools, the resulting new varieties would be considered GMO if process of generation is considered but non-GMO if the product is considered instead. But if the same alien gene were introgressed by traditional crossing approach it would be considered non-GMO under both conditions. Defining a variety as “GMO” precipitates a costly and time-consuming approval process.

**FIGURE 3:**
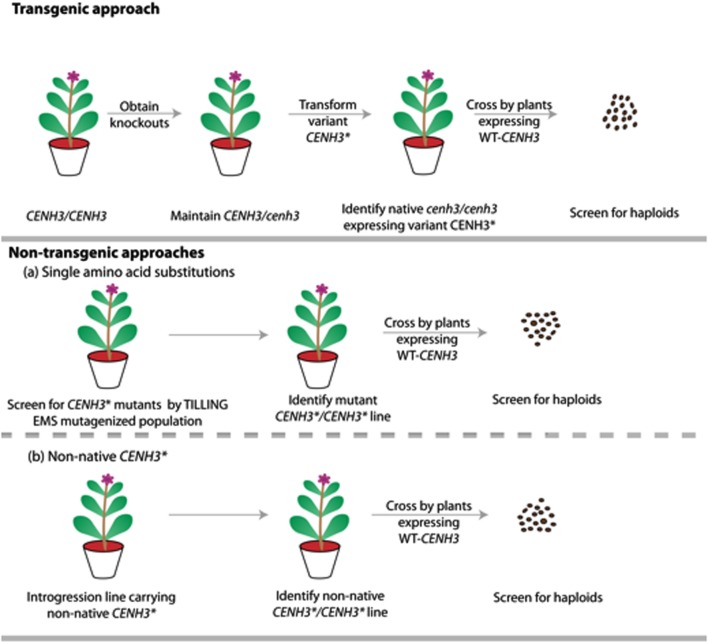
**Schematic representation comparing the steps involved in generating haploids by transgenic vs. non-transgenic approaches.** The transgenic approach involves generating *CENH3* knockouts by TILLING or genome editing, with transgenic addition of point mutant, non-native or *GFP-tailswap* alleles (*CENH3*^∗^). The non-transgenic approach involves novel alleles, created either by chemical mutagenesis or though introgression of non-native *CENH3*. This later non-native approach is hypothetical and is yet to be demonstrated in plants. *CENH3*^∗^ represents variant *CENH3* (Point mutant *CENH3/* Non-native *CENH3/ GFP-tailswapCENH3*).

Single amino acid substitutions obtained through point mutations generated by EMS is currently accepted as non-GMO under both interpretations of the law. Therefore, under existing regulations, chemically-induced point mutations of *CENH3* offer a non-transgenic process for generation of haploids. Additionally, because alien *CENH3s* or *GFP-tailswap* are only functional in a *cenH3–/–* homozygote, identification of point-mutation-based haploid inducers may be faster than other two approaches if robust TILLING populations are already available (**Figure 3**).

## Conclusion

Haploid induction technologies- and CENH3-based HI is only one among many- significantly reduces the time and labor required to derive new true-breeding varieties. Within the short period of its discovery, CENH3-mediated genome eliminations have also been shown to be useful in trait mapping (Seymour et al., 2012), reverse breeding (Wijnker et al., 2012), and a variety of other applications (Ravi et al., 2014), all of which can be applied to crop breeding. In spite of the great success with various applications in model plant *Arabidopsis*, CENH3-mediated genome elimination needs to be tested in other crop species– its application to maize, described in this volume (Kelliher et al., 2016) is very encouraging. The possible delay in implementing this technology (first published in 2010) may be due to the lack of *CENH3* knockouts in other species. The recent development of efficient CRISPR-based gene targeting conveniently addresses this issue. *GFP-tailswap* is a simple and straightforward approach for plants with well-annotated and sequenced genomes. The alternative approaches to CENH3-mediated HI may also be employed. Non-GMO point mutants can be obtained by EMS mutagenesis, and may already be present in existing populations. The recent findings of complementation and HI by CENH3 from related species (Maheshwari et al., 2015) offers an alternative non-GMO approach for species in which EMS mutagenesis is inefficient.

The mechanism, through which CENH3 modification affects competitive crosses, without affecting self-pollinations, mitosis, or meiosis, remains a mystery. The elucidation of this process will not only provide insight into CENH3 function and the mechanism of chromosome assortment, but may reveal additional targets that will, upon modification, provide the next generation of haploid inducers.

## Author Contributions

This review was written and edited by both authors.

## Conflict of Interest Statement

The authors declare that the research was conducted in the absence of any commercial or financial relationships that could be construed as a potential conflict of interest. The reviewer JK and handling Editor declared their shared affiliation, and the handling Editor states that the process nevertheless met the standards of a fair and objective review.

## References

[B1] Allshire R. C. Karpen G. H. (2008). Epigenetic regulation of centromeric chromatin: old dogs, new tricks? *Nat. Rev. Genet*. 9 923–937. 10.1038/nrg2466 19002142 PMC2586333

[B2] Buchwitz B. J. Ahmad K. Moore L. L. Roth M. B. Henikoff S. (1999). Cell division: a histone-H3-like protein in *C. elegans. Nature* 401 547–548. 10.1038/44062 10524621

[B3] Cutter A. R. Hayes J. J. (2015). A brief review of nucleosome structure. *FEBS Lett.* 589 2914–2922. 10.1016/j.febslet.2015.05.016 25980611 PMC4598263

[B4] Dunwell J. M. (2010). Haploids in flowering plants: origins and exploitation. *Plant Biotechnol. J.* 8 377–424. 10.1111/j.1467-7652.2009.00498.x 20233334

[B5] Dwivedi S. L. Britt A. B. Tripathi L. Sharma S. Upadhyaya H. D. Ortiz R. (2015). Haploids: constraints and opportunities in plant breeding. *Biotechnol. Adv.* 33 812–829. 10.1016/j.biotechadv.2015.07.001 26165969

[B6] Fleischmann A. Michael T. P. Rivadavia F. Sousa A. Wang W. Temsch E. M. (2014). Evolution of genome size and chromosome number in the carnivorous plant genus *Genlisea* (Lentibulariaceae), with a new estimate of the minimum genome size in angiosperms. *Ann. Bot.* 114 1651–1663. 10.1093/aob/mcu189 25274549 PMC4649684

[B7] Gibcus J. H. Dekker J. (2013). The hierarchy of the 3D genome. *Mol. Cell* 49 773–782. 10.1016/j.molcel.2013.02.011 23473598 PMC3741673

[B8] Guha S. Maheshwari S. C. (1964). In vitro production of embryos from anthers of datura. *Nature* 204 497–497. 10.1038/204497a0

[B9] Hartung F. Schiemann J. (2014). Precise plant breeding using new genome editing techniques: opportunities, safety and regulation in the EU. *Plant J.* 78 742–752. 10.1111/tpj.12413 24330272

[B10] Henikoff S. Smith M. M. (2015). Histone variants and epigenetics. *Cold Spring Harbor Perspect. Biol.* 7:a019364 10.1101/cshperspect.a019364 PMC4292162 25561719

[B11] Howman E. V. Fowler K. J. Newson A. J. Redward S. MacDonald A. C. Kalitsis P. (2000). Early disruption of centromeric chromatin organization in centromere protein A (Cenpa) null mice. *Proc. Natl. Acad. Sci. U.S.A.* 97 1148–1153. 10.1073/pnas.97.3.1148 10655499 PMC15551

[B12] Ingouff M. Rademacher S. Holec S. Šoljić L. Xin N. Readshaw A. (2010). Zygotic resetting of the HISTONE 3 variant repertoire participates in epigenetic reprogramming in *Arabidopsis*. *Curr. Biol.* 20 2137–2143. 10.1016/j.cub.2010.11.012 21093266

[B13] Karimi-Ashtiyani R. Ishii T. Niessen M. Stein N. Heckmann S. Gurushidze M. (2015). Point mutation impairs centromeric CENH3 loading and induces haploid plants. *Proc. Natl. Acad. Sci. U.S.A.* 112 11211–11216. 10.1073/pnas.1504333112 26294252 PMC4568683

[B14] Kasha K. J. Kao K. N. (1970). High frequency haploid production in barley (*Hordeum vulgare* L.). *Nature* 225 874–876. 10.1038/225874a0 16056782

[B15] Kelliher T. Starr D. Wang W. McCuiston J. Zhong H. Nuccio M. L. (2016). Maternal haploids are preferentially induced by CENH3-tailswap transgenic complementation in maize. *Front. Plant Sci.* 7:414 10.3389/fpls.2016.00414 PMC4814585 27066050

[B16] Kuppu S. Tan E. H. Nguyen H. Rodgers A. Comai L. Chan S. W. L. (2015). Point mutations in centromeric histone induce post-zygotic incompatibility and uniparental inheritance. *PLoS Genet.* 11:e1005494 10.1371/journal.pgen.1005494 PMC4564284 26352591

[B17] Lermontova I. Koroleva O. Rutten T. Fuchs J. Schubert V. Moraes I. (2011). Knockdown of CENH3 in *Arabidopsis* reduces mitotic divisions and causes sterility by disturbed meiotic chromosome segregation. *Plant J.* 68 40–50. 10.1111/j.1365-313X.2011.04664.x 21635586

[B18] Lermontova I. Kuhlmann M. Friedel S. Rutten T. Heckmann S. Sandmann M. (2013). *Arabidopsis* KINETOCHORE NULL2 is an upstream component for centromeric histone H3 variant cenH3 deposition at centromeres. *Plant Cell* 25 3389–3404. 10.1105/tpc.113.114736 24014547 PMC3809539

[B19] Lermontova I. Sandmann M. Demidov D. (2014). Centromeres and kinetochores of Brassicaceae. *Chromosome Res.* 22 135–152. 10.1007/s10577-014-9422-z 24801345

[B20] Liu Y. Su H. Zhang J. Liu Y. Han F. Birchler J. A. (2015). Dynamic epigenetic states of maize centromeres. *Front. Plant Sci.* 6:904 10.3389/fpls.2015.00904 PMC4620398 26579154

[B21] Maehara K. Takahashi K. Saitoh S. (2010). CENP-A reduction induces a p53-dependent cellular senescence response to protect cells from executing defective mitoses. *Mol. Cell. Biol.* 30 2090–2104. 10.1128/MCB.01318-09 20160010 PMC2863584

[B22] Maheshwari S. Tan E. H. West A. Franklin F. C. H. Comai L. Chan S. W. L. (2015). Naturally occurring differences in CENH3 affect chromosome segregation in zygotic mitosis of hybrids. *PLoS Genet.* 11:e1004970 10.1371/journal.pgen.1004970 PMC4314295 25622028

[B23] Malik H. S. Henikoff S. (2003). Phylogenomics of the nucleosome. *Nat. Struct. Mol. Biol.* 10 882–891. 10.1038/nsb996 14583738

[B24] Marshall O. J. Chueh A. C. Wong L. H. Choo K. A. (2008). Neocentromeres: new insights into centromere structure, disease development, and karyotype evolution. *Am. J. Hum. Genet.* 82 261–282. 10.1016/j.ajhg.2007.11.009 18252209 PMC2427194

[B25] Mattiroli F. D’Arcy S. Luger K. (2015). The right place at the right time: chaperoning core histone variants. *EMBO Rep.* 16:e201540840 10.15252/embr.201540840 PMC4641499 26459557

[B26] Moraes I. R. Lermontova I. Schubert I. (2011). Recognition of *A. thaliana* centromeres by heterologous CENH3 requires high similarity to the endogenous protein. *Plant Mol. Biol.* 75 253–261. 10.1007/s11103-010-9723-3 21190064

[B27] Müller S. Almouzni G. (2014). A network of players in H3 histone variant deposition and maintenance at centromeres. *Biochim. Biophys. Acta (BBA)-Gene Regul. Mech.* 1839 241–250. 10.1016/j.bbagrm.2013.11.008 24316467

[B28] Pellicer J. Fay M. F. Leitch I. J. (2010). The largest eukaryotic genome of them all? *Bot. J. Linn. Soc.* 164 10–15. 10.1111/j.1095-8339.2010.01072.x

[B29] Ravi M. Chan S. W. L. (2010). Haploid plants produced by centromere-mediated genome elimination. *Nature* 464 615–618. 10.1038/nature08842 20336146

[B30] Ravi M. Kwong P. N. Menorca R. M. G. Valencia J. T. Ramahi J. S. Stewart J. L. (2010). The rapidly evolving centromere-specific histone has stringent functional requirements in *Arabidopsis thaliana*. *Genetics* 186 461–471. 10.1534/genetics.110.120337 20628040 PMC2954480

[B31] Ravi M. Marimuthu M. P. A. Tan E. H. Maheshwari S. Henry I. M. Marin-Rodriguez B. (2014). A haploid genetics toolbox for *Arabidopsis thaliana*. *Nat. Commun.* 5:5334 10.1038/ncomms6334 25358957

[B32] Ravi M. Shibata F. Ramahi J. S. Nagaki K. Chen C. Murata M. (2011). Meiosis-specific loading of the centromere-specific histone CENH3 in *Arabidopsis thaliana*. *PLoS Genet.* 7:e1002121 10.1371/journal.pgen.1002121 PMC3111537 21695238

[B33] Sanei M. Pickering R. Kumke K. Nasuda S. Houben A. (2011). Loss of centromeric histone H3 (CENH3) from centromeres precedes uniparental chromosome elimination in interspecific barley hybrids. *Proc. Natl. Acad. Sci. U.S.A.* 108 E498–E505. 10.1073/pnas.1103190108 21746892 PMC3158150

[B34] Sekulic N. Black B. E. (2012). Molecular underpinnings of centromere identity and maintenance. *Trends Biochem. Sci.* 37 220–229. 10.1016/j.tibs.2012.01.003 22410197 PMC3614489

[B35] Seymour D. K. Filiault D. L. Henry I. M. Monson-Miller J. Ravi M. Pang A. (2012). Rapid creation of *Arabidopsis* doubled haploid lines for quantitative trait locus mapping. *Proc. Natl. Acad. Sci. U.S.A.* 4227–4232. 10.1073/pnas.1117277109 22371599 PMC3306714

[B36] Steiner F. A. Henikoff S. (2015). Diversity in the organization of centromeric chromatin. *Curr. Opin. Genet. Dev.* 31 28–35. 10.1016/j.gde.2015.03.010 25956076

[B37] Steinitz-Sears L. M. (1963). Chromosome studies in *Arabidopsis thaliana*. *Genetics* 48 483–490. 17248159 10.1093/genetics/48.4.483 PMC1210487

[B38] Stimpson K. M. Sullivan B. A. (2010). Epigenomics of centromere assembly and function. *Curr. Opin. Cell Biol.* 22 772–780. 10.1016/j.ceb.2010.07.002 20675111

[B39] Stoler S. Keith K. C. Curnick K. E. Fitzgerald-Hayes M. (1995). A mutation in CSE4, an essential gene encoding a novel chromatin-associated protein in yeast, causes chromosome nondisjunction and cell cycle arrest at mitosis. *Genes Dev.* 9 573–586. 10.1101/gad.9.5.573 7698647

[B40] Talbert P. B. Masuelli R. Tyagi A. P. Comai L. Henikoff S. (2002). Centromeric localization and adaptive evolution of an *Arabidopsis* histone H3 variant. *Plant Cell Online* 14 1053–1066. 10.1105/tpc.010425 PMC150606 12034896

[B41] Tan E. H. Henry I. M. Ravi M. Bradnam K. R. Mandakova T. Marimuthu M. P. (2015). Catastrophic chromosomal restructuring during genome elimination in plants. *eLife* 4:e06516 10.7554/eLife.06516 PMC4461816 25977984

[B42] Wang Z. Yin H. Lv L. Feng Y. Chen S. Liang J. (2014). Unrepaired DNA damage facilitates elimination of uniparental chromosomes in interspecific hybrid cells. *Cell Cycle* 13 1345–1356. 10.4161/cc.28296 24608870 PMC4049971

[B43] Wędzony M. Forster B. P. Żur I. Golemiec E. Szechyńska-Hebda M. Dubas E. (2009). “Progress in doubled haploid technology in higher plants,” in *Advances in Haploid Production in Higher Plants*, eds Touraev A. Forster B. Jain S. M. (Amsterdam: Springer), 1–33. 10.1007/978-1-4020-8854-4_1

[B44] Westhorpe F. G. Straight A. F. (2015). The centromere: epigenetic control of chromosome segregation during mitosis. *Cold Spring Harb. Perspect. Biol.* 7:a015818 10.1101/cshperspect.a015818 PMC4292168 25414369

[B45] Wieland G. Orthaus S. Ohndorf S. Diekmann S. Hemmerich P. (2004). Functional complementation of human centromere protein A (CENP-A) by Cse4p from *Saccharomyces cerevisiae*. *Mol. Cell. Biol.* 24 6620–6630. 15254229 10.1128/MCB.24.15.6620-6630.2004 PMC444843

[B46] Wijnker E. van Dun K. de Snoo C. B. Lelivelt C. L. C. Keurentjes J. J. B. Naharudin N. S. (2012). Reverse breeding in *Arabidopsis thaliana* generates homozygous parental lines from a heterozygous plant. *Nat. Genet.* 44 467–470. 10.1128/MCB.24.15.6620-6630.2004 22406643

